# A Novel Behavioral Intervention for Rural Appalachian Cancer Survivors (*we*Survive): Participatory Development and Proof-of-Concept Testing

**DOI:** 10.2196/26010

**Published:** 2021-04-12

**Authors:** Kathleen J Porter, Katherine E Moon, Virginia T LeBaron, Jamie M Zoellner

**Affiliations:** 1 Department of Public Health Sciences School of Medicine University of Virginia Christiansburg, VA United States; 2 Department of Acute & Specialty Care School of Nursing University of Virginia Charlottesville, VA United States

**Keywords:** cancer survivors, quality of life, behavior change, rural, feasibility, Appalachia

## Abstract

**Background:**

Addressing the modifiable health behaviors of cancer survivors is important in rural communities that are disproportionately impacted by cancer (eg, those in Central Appalachia). However, such efforts are limited, and existing interventions may not meet the needs of rural communities.

**Objective:**

This study describes the development and proof-of-concept testing of *we*Survive, a behavioral intervention for rural Appalachian cancer survivors.

**Methods:**

The Obesity-Related Behavioral Intervention Trials (ORBIT) model, a systematic model for designing behavioral interventions, informed the study design. An advisory team (n=10) of community stakeholders and researchers engaged in a participatory process to identify desirable features for interventions targeting rural cancer survivors. The resulting multimodal, 13-week *we*Survive intervention was delivered to 12 participants across the two cohorts. Intervention components included in-person group classes and group and individualized telehealth calls. Indicators reflecting five feasibility domains (acceptability, demand, practicality, implementation, and limited efficacy) were measured using concurrent mixed methods. Pre-post changes and effect sizes were assessed for limited efficacy data. Descriptive statistics and content analysis were used to summarize data for other domains.

**Results:**

Participants reported high program satisfaction (acceptability). Indicators of demand included enrollment of cancer survivors with various cancer types and attrition (1/12, 8%), recruitment (12/41, 30%), and attendance (median 62%) rates. Dietary (7/12, 59%) and physical activity (PA; 10/12, 83%) behaviors were the most frequently chosen behavioral targets. However, the findings indicate that participants did not fully engage in action planning activities, including setting specific goals. Implementation indicators showed 100% researcher fidelity to delivery and retention protocols, whereas practicality indicators highlighted participation barriers. Pre-post changes in limited efficacy outcomes regarding cancer-specific beliefs and knowledge and behavior-specific self-efficacy, intentions, and behaviors were in desired directions and demonstrated small and moderate effect sizes. Regarding dietary and PA behaviors, effect sizes for fruit and vegetable intake, snacks, dietary fat, and minutes of moderate-to-vigorous activity were small (Cohen *d*=0.00 to 0.32), whereas the effect sizes for change in PA were small to medium (Cohen *d*=0.22 to 0.45).

**Conclusions:**

*we*Survive has the potential to be a feasible intervention for rural Appalachian cancer survivors. It will be refined and further tested based on the study findings, which also provide recommendations for other behavioral interventions targeting rural cancer survivors. Recommendations included adding additional recruitment and engagement strategies to increase demand and practicality as well as increasing accountability and motivation for participant involvement in self-monitoring activities through the use of technology (eg, text messaging). Furthermore, this study highlights the importance of using a systematic model (eg, the ORBIT framework) and small-scale proof-of-concept studies when adapting or developing behavioral interventions, as doing so identifies the intervention’s potential for feasibility and areas that need improvement before time- and resource-intensive efficacy trials. This could support a more efficient translation into practice.

## Introduction

Cancer survivors comprise approximately 5% of the US population, and the number of cancer survivors is expected to increase by almost 30% over the next 10 years [1]. Although cancer survivors live longer, evidence suggests that they continue to engage in behaviors that increase their risk for recurrence, new cancers after treatment, and other chronic diseases that could impair survivorship outcomes [2,3]. Health behaviors that are recommended for cancer survivors to engage in include healthy diet and weight, being physically active, avoiding or stopping tobacco use, limiting alcohol consumption, and practicing sun safety [4,5]. Cancer survivors may be primed to change their health behaviors, as the cancer diagnosis and treatment may serve as *teachable moments* that motivate them to improve health behaviors. Therefore, addressing the health behaviors of cancer survivors has been identified as a priority in both clinical and community settings [6].

Addressing the health behaviors of cancer survivors is particularly important in health disparate communities, such as those in rural Central Appalachia. These communities are disproportionately impacted by cancer, as indicated by higher cancer mortality rates than those of nonrural communities [7]. There are also high rates of low educational attainment and low socioeconomic status in this region [8], and these social determinants of health are associated with a greater likelihood of engaging in unhealthy behaviors after treatment [2]. In addition, these communities often have a high prevalence of other chronic health conditions, such as type 2 diabetes, obesity, and heart disease [9-12], which can adversely impact cancer outcomes and mortality. Importantly, the development and management of these health conditions can be impacted by changing health behaviors. However, efforts to address the health behaviors of cancer survivors in Appalachia, similar to other rural areas, have been limited [13].

Increasing efforts to integrate interventions for cancer survivors that target modifiable health behaviors may be a strategic way to reduce cancer disparities in this region and others. Although there are existing behavioral interventions for cancer survivors, most of them are designed for survivors of a specific type of cancer and use one mode of delivery [14-17]. In addition, few of these existing interventions have been specifically developed for the needs of rural cancer survivors. Therefore, existing interventions would need to be adapted or a new intervention would need to be developed to meet the needs of cancer survivors in Appalachia.

Using a systematic process to develop or adapt an intervention allows for the assessment of the intervention’s potential relevance, clinical efficacy, and sustainability. This information is particularly vital for interventions that have the ultimate goal of being translated into real-world settings. The Obesity-Related Behavioral Intervention Trials (ORBIT) model presents a systematic process of translating basic and clinical behavioral science findings into behavioral interventions [18]. Although initially designed for the development of obesity-focused trials, the systematic steps of the ORBIT model are applicable for the design of behavioral interventions targeting other health conditions. This paper describes how researchers affiliated with the University of Virginia (UVA) Cancer Center and community stakeholders from its rural Appalachia catchment area in southwest Virginia employed phase 1 and phase 2 of the ORBIT model to adapt or develop and pilot test a behavioral intervention for cancer survivors.

## Methods

### Design

This two-phase mixed methods study describes the development and initial pilot testing of a behavioral intervention for rural cancer survivors. The process, guided by the ORBIT model [18] and feasibility framework by Bowen et al [19], provides a conceptual framework for the evaluation of a proof-of-concept study. The ORBIT model includes 4 phases—phase 1: define and refine basic elements, phase 2: preliminary testing, phase 3: efficacy testing, and phase 4: effectiveness testing. This study focused on the first 2 phases. The feasibility framework by Bowen et al [19] identifies 8 key domains to measure during feasibility trials at both the participant and organizational levels. This study measures indicators for the 5 domains that are appropriate for the early proof-of-concept trial phase: acceptability, demand, implementation, practicality, and limited efficacy testing. The domains are listed in Table 1.

**Table 1:** Summary of measures used in the feasibility trial of *we*Survive.

Feasibility domain, definition, indicator, and measure			Baseline	Postassessment	Process evaluation
**Acceptability: extent to which the intervention is judged as suitable, satisfying, or attractive to recipients**					
	**Organizational perceptions**				
		Recruitment memos	—^a^	—	✓^b^
	**Participant satisfaction**				
		Summative evaluation	—	✓	—
**Demand: extent to which the intervention is likely to be used**					
	**Organizational adoption**				
		Recruitment memos	—	—	✓
	**Recruitment rates**				
		Recruitment logs	—	—	✓
	**Participant engagement**				
		Attendance logs	—	—	✓
		Class and call memos	—	—	—
		Class or call artifacts	—	—	—
	**Behavioral target chosen by participants**				
		Summative evaluation	—	✓	✓
		Class or call artifacts	—	—	—
**Practicality: extent to which the intervention can be carried out with intended participants using existing means, resources, and circumstances and without outside intervention**					
	**Barriers and facilitators of participant engagement**				
		Summative evaluation	—	✓	—
**Implementation: extent the intervention can be successfully delivered to intended participants**					
	**Recruitment execution**				
		Recruitment memos	—	—	✓
		Recruitment logs	—	—	—
	***we*Survive delivery**				
		Class or call memos	—	—	✓
**Limited efficacy: the promise of the intervention to be successful with the intended population**					
	**Changes in cancer-related beliefs**				
		Cancer belief questions from HiNTS^c^	✓	✓	—
	**Changes in diet and physical activity self-efficacy**				
		Scaled survey questions	✓	✓	—
	**Changes in diet and physical activity intentions**				
		Scaled survey questions	✓	✓	—
	**Changes in dietary behaviors**				
		NCI^d^ multifactor screener	✓	✓	—
	**Changes in physical activity behaviors**				
		Modified Godin	✓	✓	—
		L-CAT^e^	—	—	—
	**Changes in social network size**				
		Cancer survivor social networks measure	✓	✓	—
	**Changes in quality of life**				
		Quality of life patient or cancer survivor version	✓	✓	—

^a^Related data were not collected.

^b^Related data were collected.

^c^HiNTS: Health Information National Trends Survey.

^d^NCI: National Cancer Institute.

^e^L-CAT: Stanford Leisure-Time Activity Categorical Item.

### ORBIT Model Phase 1: Define and Refine Basic Elements

#### Intention of Phase

The purpose of phase 1 of the ORBIT model is to develop a hypothesized pathway through which behavioral intervention could impact health and determine components, duration, mode of delivery, and tailoring needs [18]. For our study, the intention for this phase was to identify and adapt an existing intervention or, if needed, develop a novel intervention using best practices. We approached this phase by (1) conducting literature searches and (2) engaging an advisory team of local stakeholders in a participatory development process.

#### Literature Search

We conducted a search of those listed in the National Cancer Institute’s (NCI) Research Testing Intervention/Program website [20] and through PubMed to identify existing behavioral interventions for cancer survivors. The identified interventions were reviewed during participatory processes.

#### Participatory Process

This process was guided by a comprehensive participatory planning and evaluation process [21] (described below). It incorporated the Putting Public Health Evidence in Action training [22] and focused on the sessions related to identifying, selecting, and adapting evidence-based interventions.

To recruit advisory team members, the study was presented to all members of the Cancer Center Without Walls Southwest Virginia Community Advisory Board (CAB) during a quarterly CAB meeting. The CAB consists of representatives from local health care systems and other organizations that work on cancer-related issues, community members, and the UVA Cancer Center faculty and staff. The CAB members who were interested in joining the advisory team contacted the research team. The resulting advisory team consisted of 10 members: 6 community stakeholders, 1 UVA Cancer Center Outreach and Engagement staff member, and 3 interdisciplinary UVA faculty members with expertise in behavioral interventions, oncology, and community engagement. Community stakeholders represented local health systems (n=2), the social services sector (n=2), and higher education (n=2). The 3 members were cancer survivors.

The advisory team engaged in 6 meetings over 6 months, three 1-hour in-person meetings, and three 1-hour conference calls. The intention of these meetings was to identify key recommendations for what the intervention should address and to use these recommendations to identify and either adapt or develop a behavioral intervention. Planned activities included sharing previous experiences with behavioral interventions for cancer survivors and perceptions of needed and acceptable components, reviewing and commenting on existing behavioral interventions for cancer survivors, and deciding upon the intervention and identifying adaptations. Notes and reflection worksheets completed during meetings were reviewed, summarized, and used to identify key action steps between meetings. During this process and based on the literature review, it became evident that existing interventions did not meet local needs and that a novel intervention would need to be developed.

**Figure 1:**
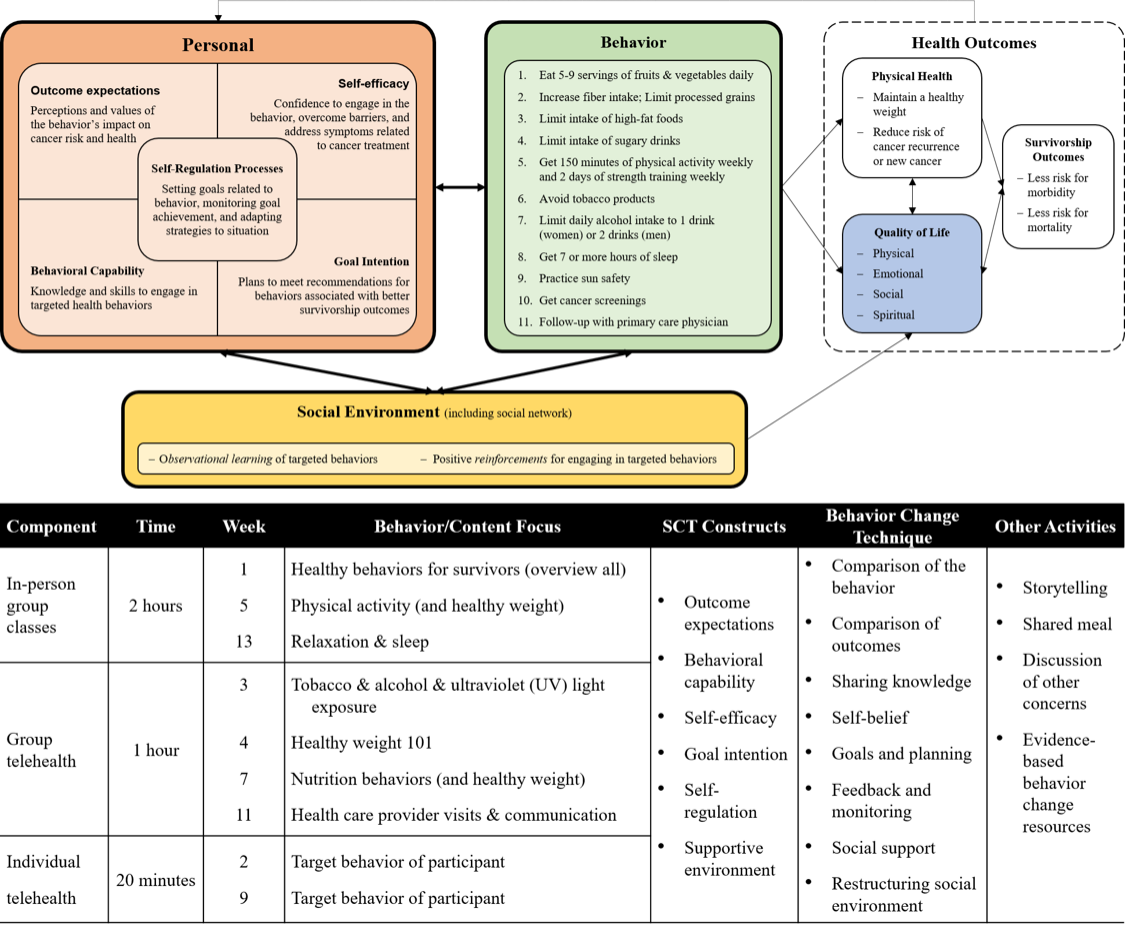
*we*Survive program conceptual model and component design. SCT: Social Cognitive Theory.

Through the participatory process, the advisory team identified 4 key recommendations that an ideal behavioral intervention for rural Appalachian cancer survivors would need to take into account: (1) incorporation of both in-person and telehealth components so that participants could engage even if they had barriers to one delivery mode; (2) utilization of strategies that promoted action planning and storytelling; (3) addressing multiple behaviors; and (4) opening the program to all adult cancer survivors regardless of gender or cancer type. A conceptual model and program design were developed using these recommendations and a review of the best practices (Figure 1).

The resulting intervention, *we*Survive, was rooted in Social Cognitive Theory (SCT) [23] and targeted improving participant quality of life (QoL) through the improvement of 11 health behaviors associated with better cancer survivorship outcomes, including dietary and physical activity (PA) behaviors (Figure 1) [4,5]. Participants self-selected 1 or 2 behaviors they wanted to focus on in the first in-person group class. To make this selection, participants engaged in a guided reflection through which they assessed their level of engagement with each healthy behavior, whether they wanted to improve upon it, and their confidence in making the improvements or changes.

Participants received 10 hours of contact over 13 weeks. There were 3 in-person group classes, 4 group telehealth calls, and 2 individualized telehealth calls. Telehealth activities were assessed using Zoom (Zoom Video Communications Inc) [24]. Each component was led by KP. The activities in each component addressed 6 SCT constructs: outcome expectations, behavioral capability, self-efficacy, goal intention, self-regulation, and supportive environment [23]. Behavior change techniques, including self-monitoring [25], that tapped into the theory constructs and addressed aspects of QoL were included in each component. To support the execution of the components and behavior change, participants received a physical workbook that included class and call content, action planning materials, and evidenced-based resources (eg, exercise DVDs). Group components also provided avenues for discussion about participants’ experiences as a cancer survivor to extend social networks to include other cancer survivors.

### ORBIT Framework Phase 2: Preliminary Testing

#### Intention of Phase

The goal of phase 2 of the ORBIT model is to determine the potential of the intervention to produce clinically significant findings and evaluate intervention feasibility. A hallmark of this phase is the establishment of a clearly articulated intervention protocol (eg, curriculum, protocols for recruitment, retention, and data collection). This phase consists of proof-of-concept studies, followed by pilot studies. Proof-of-concept studies aim to determine whether the intervention warrants more rigorous testing or whether modifications are needed before additional testing. Proof-of-concept studies are usually conducted using quasi-experimental designs and usually have small sample sizes. Small sample sizes are acceptable, as the intention is to identify clinically significant impacts, not statistically significant ones.

The *we*Survive proof-of-concept study used a single-group pre-post design and a concurrent mixed methods approach [26]. All study procedures were approved by the UVA Institutional Review Board (IRB). As study measures were completed over the telephone to reduce participant burden, participants provided verbal informed consent. They received US $25 in gift cards to complete each of the baseline assessments and postassessments. Participants also received a US $5 gas card for each in-person class attended to assist with cover transportation costs.

#### Recruitment

Recruitment strategies were executed at the organizational and participant levels. At the organizational level, 2 local health care organizations that provide clinical care to cancer survivors were approached to be a part of this study. Importantly, a member of the advisory team worked for one of these organizations. To recruit the organizations, we presented the intention and design of the *we*Survive intervention and the proof-of-concept trial to key clinical staff. After the organizational staff expressed interest, we reviewed the participant recruitment protocol with them and tailored the recruitment strategy, including a communication plan, to their needs. As needed, we obtained approval from the IRB of the organizations.

Following organization recruitment, *2* cohorts of participants were recruited from 2 recruited organizations. To be eligible, participants had to be cancer free, had to have completed primary treatment within the past 5 years, and be English speaking. Inclusion was not limited by cancer type or gender. The initial recruitment protocol involved selecting clinical staff who interacted with cancer survivors during their follow-up appointments to directly present the *we*Survive intervention to eligible survivors and solicit their interest. Then, for interested survivors, the clinician would securely share their contact information with the research team or show the prospective participant how to contact us. This strategy was expanded to include other active (eg, direct communication with research staff during follow-up appointments, booths at survivorship dinners, Relay-4-Life events) and passive (eg, flyers in waiting rooms) recruitment strategies.

#### Data Collection and Measures

Participant-level data were collected at baseline and postassessment. Process data were collected during the execution of the proof-of-concept trial. Table 1 describes the measures used to assess the indicators for the assessed feasibility domains.

During recruitment, research and organizational staff maintained recruitment logs and kept recruitment memos of interactions with prospective participants. These logs included the gender, age, and decision of all prospective participants with whom staff members spoke about joining *we*Survive as well as where and by whom they were approached. The research staff also kept notes during meetings with the organizational staff.

Research staff maintained attendance logs*,* recording attendance for each component.

Class artifacts, including action plans during the first group class, were photographed. The research staff also kept delivery memos of how each component went and the completeness of each activity. Tracking sheets were also used to monitor adherence to the intervention protocols (eg, sending reminder messages, contacts for individual calls).

To measure limited efficacy measures, participants completed a survey packet at baseline and postintervention. The packet was completed over the phone with a trained research staff member. The included measures were validated, cancer survivor specific, and/or successfully used in the region before. A total of 2 questions from the Health Information National Trends Survey were used to identify beliefs about cancer [27]. Single-item questions were used to assess self-efficacy and behavioral intentions to change dietary and PA behaviors [28]. The targeted dietary and PA health behaviors were assessed using scales from the NCI Multifactor Screener [29], Stanford Leisure-Time Activity Categorical Item (L-CAT) [30], and modified Godin [28]. Although behaviors, intentions, and self-efficacy were also assessed for other health behaviors, they were not reported in this paper because of the infrequency with which they were selected by participants. The Cancer Survivor Social Networks Measure [31] was used to assess participants’ social networks. QoL was measured using the Quality of Life Patient/Cancer Survivor version [32]. Additional details regarding the measures can be found in Table 2.

Following completion of the postassessment survey, participants completed a *summative evaluation*. This semistructured interview assessed indicators of acceptability (ie, satisfaction), demand (ie, chosen behavioral target, reasons for choosing the behavioral target), and practicality (ie, barriers and facilitators of attendance) [33].

Participant *demographics* (ie, gender, age, race or ethnicity, income, educational attainment) and cancer experience (ie, type, staging, type of treatment, date of primary treatment completion) were collected at baseline. Health literacy was also measured using a validated 3-item brief questionnaire [34].

**Table 2:** Limited efficacy-related outcomes.

Variable type and specific variable		Scale	Preassessment (n=11), mean (SD)	Postassessment (n=11), mean (SD)	Direction of change	*t* statistic (*P* value)	Cohen *d*
**Cancer beliefs and knowledge**							
	There are so many recommendations about preventing cancer, it's hard to know which ones to follow	5-point Likert scale (1=strongly disagree; 5=strongly agree)	4.0 (1.34)	3.6 (1.51)	↓^a^	1.102 (.30)	−0.28
	Cancer is most often caused by a person's behavior or lifestyle	5-point Likert scale (1=strongly disagree; 5=strongly agree)	2.6 (1.63)	3.3 (1.62)	↑^b^	1.295 (.22)	0.43
**Self-efficacy**							
	Self-efficacy to eat 5-9 servings of fruits and vegetables a day	10-point Likert scale (1=not at all confident; 10=totally confident)	6.7 (2.65)	6.6 (1.63)	↓	0.118 (.91)	−0.05
	Self-efficacy to eat a diet with less saturated fat	10-point Likert scale (1=not at all confident; 10=totally confident)	7.6 (1.92)	7.4 (1.96)	↓	0.319 (.76)	−0.10
	Self-efficacy to be physically active for 150 min a week	10-point Likert scale (1=not at all confident; 10=totally confident)	6.5 (3.39)	6.8 (2.79)	↑	0.498 (.63)	0.10
**Behavior-specific intentions**							
	Eat 5-9 servings of fruits and vegetables a day	5-point scale (1=no intention to engage in at all; 5=already doing)	3.2 (1.40)	3.6 (1.29)	↑	1.174 (.27)	0.30
	Eat a diet with less saturated fat	5-point scale (1=no intention to engage in at all; 5=already doing)	3.9 (1.38)	4.0 (1.18)	↑	0.289 (.78)	0.08
	Be physically active for 150 min a week	5-point scale (1=no intention to engage in at all; 5=already doing)	3.2 (1.32)	3.9 (1.14)	↔^c^	2.667 (.02)	0.57
**Health behaviors**							
	Fruit and vegetables	Daily portions	1.8 (1.38)	1.8 (.92)	↔	0.096 (.93)	0.00
	Snack foods	Daily portions	1.1 (.84)	1.0 (1.58)	↓	0.178 (.86)	−0.08
	Dietary fat	Daily portions	5.3 (6.53)	3.5 (4.45)	↓	1.402 (.19)	−0.32
	Moderate-vigorous physical activity	Minutes per week	115.0 (137.20)	158.6 (237.78)	↑	0.889 (.40)	0.22
	Self-reported frequency of physical activity	6-point scale (1=very little physical activity; 6=30 min of vigorous activity 5 or more times a week)	2.4 (.84)	2.8 (.92)	↑	1.809 (.10)	0.45
**Social network**							
	Cancer-specific social support network size	Score of 0-15	9.6 (2.01)	10.5 (2.50)	↑	1.423 (.19)	0.40
**Quality of life**							
	Overall	11-point Likert scale (0=extremely negative; 10=extremely positive)	8.1 (1.39)	7.8 (1.78)	↓	1.055 (.32)	−0.19
	Physical	11-point Likert scale (0=extremely negative; 10=extremely positive)	8.6 (1.47)	7.8 (2.43)	↓	1.173 (.27)	−0.40
	Emotional	11-point Likert scale (0=extremely negative; 10=extremely positive)	7.7 (1.77)	7.3 (2.26)	↓	1.303 (.22)	−0.20
	Social	11-point Likert scale (0=extremely negative; 10=extremely positive)	7.8 (2.40)	7.6 (2.33)	↓	0.578 (.58)	−0.08
	Spiritual	11-point Likert scale (0=extremely negative; 10=extremely positive)	8.4 (1.74)	8.4 (2.17)	↔	0.120 (.91)	0.00

^a^Decrease in score from pre to postassessment.

^b^Increase in score from pre to postassessment.

^c^No change in score from pre to postassessment.

#### Data Analysis

Descriptive statistics (frequencies, means, medians, and ranges) were used to summarize participant demographics, participant satisfaction, recruitment and engagement rates, and selected behavioral targets. Limited efficacy measures were scored using standard procedures, and paired, two-tailed *t* tests were used to compare baseline and posttest responses for limited efficacy measures for program completers (n=11). Cohen *d* was calculated for each limited efficacy outcome. Open-ended data related to participant satisfaction, facilitators and barriers to engagement, component execution, and perceptions of organizations were content coded by one researcher and reviewed by another. Quantitative and qualitative data for each indicator were triangulated [26].

## Results

### Participants

A total of 12 participants were enrolled in 2 sequential pilot cohorts (n=5 and n=7). The participants were 75% (8/12) female and 100% (12/12) White. The average age of participants was 64 (SD 6.37) years, and 75% (9/12) were married. Half (6/12, 50%) of the participants were employed full-time, 33% (4/12) had a high school degree or less, and 25% (3/12) made under US $25,000 a year. All participants had medical insurance, either private (5/12, 42%) or Medicare (7/12, 58%). The majority of the participants (n=11) had adequate health literacy.

The participants were survivors of 6 types of cancer: breast (6/12, 50%), prostate (3/12, 25%), skin (2/12, 17%), colon (1/12, 8%), cervical cancer (1/12, 8%), and large B-cell lymphoma (1/12, 8%). Two participants (2/12, 17%) had multiple cancers. The participants had completed chemotherapy (8/12, 67%), radiation (5/12, 42%), surgery (8/12, 67%), and stem cell treatment (1/12, 8%). Over half of the participants (7/12, 58%) received multiple treatment types. On average, participants had completed primary treatment for 13.8 months (SD 13.5; range 1-40 months) before joining the trial.

### Feasibility Indicators

The outcomes for acceptability, demand, practicality, and implementation are presented in Table 3, whereas limited efficacy outcomes are presented in Table 2.

**Table 3:** Findings related to the feasibility domains of acceptability, demand, practicality, and implementation.

Feasibility domain and indicator		Quantitative findings	Qualitative findings
**Acceptability**			
	Organizational perceptions	—^a^	Staff from the 2 organizations that were approached to host *we*Survive felt it would be beneficial to their patients
	Participant satisfaction	Overall rating, mean 10.0/10.0 (SD 0.00) Group classes, mean 9.7/10.0 (SD 0.65) Group calls, mean 9.5/10.0 (SD 0.87) Individualized calls, mean 9.7/10.0 (SD 0.53)	Perceived program benefits: Knowledge gained Opportunity to share their experiences and learn about others’ experiences Felt the program was an important wakeup call Saw the program as an opportunity to improve their lives or give back to others No facets of the program identified as “unacceptable”
**Demand**			
	Organizational adoption	The 2 (100%) health care organizations approached agreed to take part in the *we*Survive proof-of-concept trial	—
	Recruitment rates	Recruitment rate=30% (12/41) 59% (17/29) of nonenrolment was due to lack of ability to follow up with prospective participant to schedule or complete the survey 38% (11/29) of nonenrolment was due to lack of interest	—
	Participant participation	Attrition=8% (1/12) Overall attendance: median 62% (average 56%): Group class attendance: median 84% (average 72%) Group call attendance: median 50% (average 42%) Individual call attendance: median 50% (average 50%) Of the 8 participants who attended group calls, only 3 (38%) used the video portion of the telehealth platform	When completing action plans, participants often only partially completed them or just discussed their plans without writing them down. Participants appeared hesitant to set SMART^b^ goals During individual calls, 3 participants asked for and received support for specific dietary matters beyond what was in the standard curriculum
	Behavioral target chosen by participants	100% (12/12) selected diet or PA^c^: 83% (10/12) selected PA 59% (7/12) selected a dietary behavior 42% (5/12) chose both PA and diet 50% (6/12) chose a behavior other than diet or PA: sleep (3/12, 25%), stress reduction (4/12, 33%)	Reasons for choosing behaviors: Priority for personal or disease-specific reasons Perceived as easier to address
**Practicality**			
	Barriers and facilitators of participant engagement	—	Barriers to attendance: Personal and work obligations Facilitators of attendance: Participants found reminder texts helpful Expanded texting reminder system in cohort 2 to include reminder day before and 2 hours before
**Implementation**			
	Recruitment execution	Eligible participants (n=41) were approached through office visits (25/41, 61%), community events (13/41, 32%), and word of mouth (3/41, 7%): 100% of office visit referrals were executed jointly by site and research staff 100% of community event referrals were completed by research staff 100% of word-of-mouth referrals were completed outside of the clinic by site staff	At one of the 2 sites, a provider (MD or NP) introduced *we*Survive to an eligible participant. If the participant was interested, they invited the research team member to come in to speak with the participant. This process did not occur at the other site due to the distance to the site and inconsistent communication between research and site staff Organization staff were very interested in the idea of the program but were unable to follow the recruitment protocol on their own (ie, refer eligible patients without the presence of a research team member) but were able to execute when working in conjunction with research staff
	*we*Survive delivery	100% fidelity (of researchers) to the execution of intervention components and the participant retention protocol	—

^a^Quantitative or qualitative data was not collected for the feasibility domain indicator.

^b^SMART: specific, measurable, attainable, relevant, time-based.

^c^PA: physical activity.

#### Acceptability

Participants who completed the intervention (n=11) reported high satisfaction with the program (mean 10, SD 0.0) and with the individual components: group classes (mean 9.7, SD 0.65), group calls (mean 9.5, SD 0.87), and individual calls (mean 9.7, SD 0.53). Participants described benefits related to knowledge attainment, feeling that *we*Survive was a *wakeup call* to improve their health, sharing their cancer experiences and hearing others’ cancer experiences, and knowing that by being in the trial they were helping future cancer survivors. In addition, staff from the participating organizations expressed positive reactions to the program and viewed it as having the potential to be beneficial to their patients.

#### Demand

The 2 local health care organizations approached to participate in the proof-of-concept trial agreed to participate. The participant recruitment rate for the trial was 30%, with 12 of 41 eligible individuals approaching enrolment in the program. Among individuals who did not enroll, 38% (11/29) expressed a lack of interest in the program or prohibitive barriers (eg, language difficulties, transportation) and 59% (17/29) had barriers that limited scheduling surveys or completed the web-based presurvey.

Intervention attrition for the program was low, with only 1 participant (1/12, 8%) not completing the program. The median participation rate for all activities was 62%, with the medians for class, group call, and individual being 84%, 50%, and 50%, respectively. Of the 8 participants who completed group calls, only 3 (38%) used the video portion of the telehealth platform. The other 5 called into the platform using the telephone number and did not use the phone, tablet, or computer application that would have allowed for video.

Research staff noted that participants did not fully engage in self-monitoring activities, such as setting a specific behavioral goal and writing SMART (specific, measurable, attainable, relevant, time-based) goals, even with prompting. For example, a participant would broadly describe their target behavior (ie, “eat healthy” instead of “eat 5 fruits and vegetables 3 days a week day”) and would not include a plan for how they would make the change.

Although participants could choose among 11 behaviors, 100% chose either a diet (7/12, 59%) or PA behavior (10/12, 83%) and 42% (5/12) chose both. Of the 6 nondiet or PA behaviors, only 2 were selected: stress (n=3) and sleep (n=4).

#### Practicality

Participants identified personal and work obligations as their primary barriers to participate in intervention activities. They identified the reminder texts as facilitators of attendance.

#### Implementation

Staff from both organizations were unable to follow the original recruitment protocol and did not refer participants to the program without on-site support from the research staff. Therefore, it was necessary to adapt the recruitment protocol to provide on-site research staff support at the clinic and recruit through community events. Eligible participants were identified in 3 ways: during office visits (25/41, 61%), at community events (13/41, 32%), and word of mouth (3/41, 7%). Organizational staff made all word-of-mouth referrals, whereas research staff made referrals through community events. All office visit referrals occurred with the organizational and research staff working together. Organizational staff would introduce *we*Survive to an eligible participant and, if interested, a research team member provided further detail and collected their contact information to complete the surveys.

There was 100% fidelity to the delivery and retention protocols by the research staff. All planned activities for the components were executed as designed, and participant retention strategies (eg, reminder texts) were adhered to as intended.

#### Limited Efficacy

Regarding behavior-related psychosocial variables, participants changed their beliefs about cancer with respect to knowing which recommendations to follow (Cohen *d*=0.28) and the impact of lifestyle behaviors on cancer risk (Cohen *d*=0.43) in the desired direction. Self-efficacy to meet the PA guidelines changed in the desired direction, whereas changes in self-efficacy to reduce dietary fat and increase fruits and vegetables were in the undesired direction (ie, lower self-efficacy). The effect sizes for the behavioral self-efficacy variables were very small (≤0.10). Although not statistically significant, behavioral intentions to eat more fruits and vegetables, eat less fat, and meet PA guidelines changed in the desired direction. The change in intentions specific to PA was statistically significant (*P*=.02) and demonstrated a medium effect size (Cohen *d*=0.57).

Baseline to postassessment changes in dietary and PA behaviors were in the desired directions but were not statistically significant. Effect sizes for fruit and vegetable intake, snack foods, dietary fat, and minutes of moderate-vigorous activity were small (Cohen *d*=0.00 to 0.32), whereas the effect size for L-CAT score was medium (Cohen *d*=0.45).

Participants’ social networks specific to their cancer support networks increased. Although not significant, this change had a small-to-medium effect size of 0.40.

Regarding QoL indicators, there were nonsignificant decreases (ie, worsening of QoL) in all indicators. The magnitude of these changes was small for overall QoL, emotional QoL, social QoL, and spiritual QoL (Cohen *d*=0.00 to 0.20); However, the change in physical QoL from baseline to postassessment was small or medium (Cohen *d*=0.40).

## Discussion

### Principal Findings

Taken together, our results suggest that the *we*Survive intervention has the potential to be feasible. Our findings also highlight how the design and execution of the intervention and its components could be improved to further enhance its feasibility, including increasing efficacy among cancer survivors. Furthermore, outcomes also provide support for using a participatory process and a systematic planning model, such as the ORBIT model, to inform the design of behavioral interventions for cancer survivors.

### Implications for *we*Survive’s Feasibility

Our findings suggest high feasibility related to indicators of acceptability (ie, high satisfaction), demand (ie, high adoption rate by organizations, diversity of cancer survivors by cancer type and gender, low attrition rate, recruitment, and component engagement rates similar to other behavioral interventions for rural participants and/or cancer survivors [28,35-38]), and implementation (ie, high researcher fidelity to protocols). However, findings related to indicators of practicality (eg, consistent barriers to participation), implementation (eg, ability of organizational staff to follow intended delivery, retention, and recruitment protocols), and limited efficacy highlight opportunities to adjust aspects of the intervention design and delivery protocols that could improve feasibility.

Although our results do not fully confirm the feasibility of *we*Survive, they identify areas where modifications to *we*Survive’s design and protocols could strengthen feasibility. As proof-of-concept studies focus on the feasibility of the intervention, the evidence collected provides integral preliminary data not only about its clinical efficacy but also its relevance and potential sustainability. This preliminary evidence can help build an intervention that is both effective and more readily translated into practice. This is particularly important for behavioral interventions for rural cancer survivors, as efforts to address the health behaviors of cancer survivors in rural regions are limited [13].

### Recommendations to Improve Feasibility of *we*Survive and Other Behavioral Interventions for Rural Cancer Survivors

A total of 6 recommendations that impact all measured feasibility domains from this proof-of-concept study were identified. In addition to being directly relevant to the *we*Survive intervention, many of these recommendations are broadly applicable and can be used to inform future behavioral interventions for cancer survivors.

#### Tighten the Behavioral Focus of *we*Survive (Demand and Efficacy)

Including a wide array of behaviors important for positive survivorship outcomes was suggested by the advisory team to ensure the applicability of the program to regional cancer survivors. However, demand findings clearly demonstrated that diet and PA were the most popular choices, with all participants choosing one or the other. In addition, limited efficacy outcomes suggest that *we*Survive impacted these behaviors and related psychosocial variables in the desired direction, with some of the PA outcomes having small-to-moderate effects. Making this adjustment would streamline *we*Survive’s behavioral focus, potentially impacting the magnitude of effects for the targeted behaviors. Although the recommendation to include a variety of behaviors may have hindered feasibility, incorporating this suggestion from the advisory team during this initial phase allowed us to better ascertain the wants of regional cancer survivors. Importantly, although the behavioral focus of *we*Survive will shift to energy-balance–related behaviors, the program will still include content related to stress reduction and sleep.

#### Add Additional Recruitment Strategies (Demand)

Although we recruited a diverse group of participants with regard to gender and cancer experience, the overall group sizes were small, and the recruitment rate of 30% was modest. During the trial, we added and adapted strategies to maximize the recruitment efforts. Successful strategies included having an on-site research staff recruit in tandem with organizational staff and promoting *we*Survive at community events targeting cancer survivors. For future trials of *we*Survive, these strategies should be incorporated into recruitment from the start.

An additional recruitment strategy was to promote *we*Survive during survivorship care plan meetings. Survivorship care plans are a highly recommended part of survivorship care [39], and more clinics are systematically using them. Suggestions for behavioral changes may be included [40], but not all clinics have the resources to facilitate behavioral changes, including those related to diet, PA, and weight change behaviors. Therefore, aligning *we*Survive with cancer care survivorship plans could make the intervention more relevant for organizations and provide a natural place for it within the workflow, which could motivate organizational staff to promote *we*Survive. Although this seems to be a logical connection, few known behavioral interventions for cancer survivors reported tying their intervention in survivorship care plans [41]. If future behavioral interventions were designed to address needs highlighted by their participants’ survivorship care plans, this could increase the demand for the program from both the participant and organizational sides and could help cancer survivors better execute their plans.

Recruitment into behavioral interventions can be one of the most difficult aspects of executing an intervention, and underaccrual of participants hinders many interventions. Past lifestyle interventions for cancer survivors have reported a range of recruitment rates ranging from 4% to 70%. Although this difficulty is prevalent in densely populated regions, it may be even greater in rural regions, such as Appalachia, which have smaller populations and lack large academic medical centers and large cancer centers. Therefore, using preliminary data to create a tailored, adaptable, and multi-faceted approach to recruitment may aid in the successful recruitment of other behavioral interventions as well [42].

#### Incorporate Strategies to Support Program Engagement (Demand and Practicality)

The participation rates from our trial were similar to those of other behavioral interventions for cancer survivors [28,35-38]. However, these rates can be improved by addressing the barriers to attendance identified by the participants (eg, conflicts with personal and work scheduling, forgetting). Future strategies include (1) having at least 2 formal day or time opportunities to participate in all group activities, (2) sending reminder texts the day before and 2 hours before the scheduled call time for virtual components, and (3) offering virtual makeup sessions. These changes could improve feasibility related to participant perceptions of acceptance and practicality for *we*Survive and could be applicable strategies for similar interventions.

In addition to overall participation rates, findings show that engagement with the video portion of the teleconferencing platform was underused. Most of the 8 participants who attended at least one group call only used the audio capabilities of the platform (82.5%), and none of the participants used the video feature for all group calls they completed. We suspect that reasons include unfamiliarity with the technology and poor internet or cellular access and/or quality. During the proof-of-concept trial, participants received a written instruction sheet, and the researcher delivered the first group class talked through the instructions. Additional activities to encourage use could include a platform demonstration, a formal system for troubleshooting barriers to using the teleconferencing platform, and structured conversations about the benefits of participation in virtual components. Providing this additional support may be valuable for rural participants in lifestyle programs, as previous studies have shown that they may hesitate to use teleconferencing platforms due to low digital literacy, privacy concerns, and fear that it might limit group connection [43]. Importantly, as found in other studies with rural populations, the video portion of teleconferencing calls enabled participants to experience greater engagement and feelings of support than they would have if these components were absent [43]. Importantly, as this study was conducted before the COVID-19 pandemic, during which the general public started regularly using Zoom and other teleconferencing platforms, this experience may make future participants more comfortable with the video feature.

#### Improve Engagement in Behavioral Self-Monitoring Strategies by Creating More Accountability and Motivation (Implementation)

Behavioral self-monitoring encompasses vital behavior change techniques, such as goal setting and self-monitoring activities, which are linked to better behavioral changes [44]. Action planning, sharing goals, and discussing progress and struggles were included in each component of *we*Survive. However, participants in this trial did not fully engage in self-monitoring activities, particularly action plans. The behavior change literature suggests that this is common and that strategies can be employed to increase engagement with action planning, such as sending motivational messages, sending text messages or email reminders, and providing feedback [45,46]. In this proof-of-concept trial for *we*Survive, personalized approaches to keep participants motivated toward and accountable for their goals were not included, as our focus was on solidifying the curriculum content and recruitment, retention, and data collection protocols. Adding accountability structures appropriate to rural populations could increase engagement with behavioral self-monitoring activities. It might also be necessary to create norms within the group activities to make participants feel comfortable to share their goals, progress, and struggles and to help one another troubleshoot their issues. Employing this recommendation will not only increase the implementation of behavioral self-monitoring activities but also limit the behavioral targets of the program and impact the intervention’s efficacy on behavioral outcomes and QoL.

#### Capture the Overall Health Experiences of the Participants During the Trial Timeline

For this proof-of-concept study of *we*Survive, there were no statistically significant yet undesired changes in QoL indicators. This undesired change is not unusual, as postassessment scores on QoL measures sometimes go in the *wrong* direction due to participants rating themselves higher at baseline, potentially because they are primed to have higher expectations for QoL. In addition, through informal conversations with participants, we learned that 3 of them had substantial negative health experiences unrelated to the trial (ie, hospitalization, injury that required surgery, negative reaction to adjuvant therapy). When they were removed from the analyses, the changes either moved in the desired direction or the magnitude of the undesired changes was reduced. If captured systematically during interventions, these participant experiences could be factored into the actual outcome analyses or provide context to their interpretation. This will allow for more context from which to interpret QoL outcomes and identify whether they are unintended consequences of the intervention.

#### Use a Participatory Process to Engage Stakeholders During Intervention Development or Adaptation Interventions (Demand)

Engaging stakeholders identified the key features that aided feasibility. Features identified by the *we*Survive advisory team impacted indicators of demand and included suggestions to blend group and individual activities and were not limited by cancer type or gender. In addition, these considerations informed the decision to measure social networks, which were found to moderately, though not significantly, increase. Interestingly, 4 of the 5 participants who did not include survivors or support groups as part of their network at baseline did at postassessment. This measurement of social networks along with broad inclusion criteria added innovative features to *we*Survive, which may aid in its future translation to practice. Although there is evidence that stronger social networks are linked to improved cancer survivorship outcomes [47] and that rural cancer survivors may be less connected than survivors in other regions [48], measuring and seeking to enhance social networks is not a common feature of behavioral interventions for cancer survivors. In addition, the advisory team recommended that the intervention allow participants to have authentic opportunities to share their stories and hear from others. Although storytelling is a noted cultural tradition in Appalachia [49] and has been used in cancer-focused interventions to transfer knowledge and address emotional and existential or spiritual concerns [50], it most likely would not have been included at this early stage of development of *we*Survive if not for the advisory team. Finally, our first site was identified by one of our community stakeholders. Stakeholder participation can strengthen the design and execution of behavioral interventions by identifying unique needs or resources within the community. Although not all the comments from the advisory team aided feasibility (ie, focusing on multiple behaviors), without our stakeholder’s input and support, many of these other features would not have been included.

### Limitations

When interpreting this study’s conclusions, it is important to consider these limitations. The participant sample for the proof-of-concept trial was small. Although this impacts statistical power and interprets limited efficacy outcomes, it was still adequate to identify effect sizes and inform other feasibility indicators. The sample was not racially diverse; however, the racial makeup of the study reflects the geographical region, which is approximately 95% non-Hispanic White [8]. In addition, the sample was diverse in terms of gender and cancer experience and represented an underserved rural population. Finally, data were primarily collected at the participant level and, as such, findings are limited to feasibility at the organizational level. Future trials of *we*Survive will need to include a more robust evaluation of organizational-level indicators, including acceptability, practicality, and feasibility at this level and the potential for integration and penetration [19], to more fully understand

feasibility and identify modifications to protocols, particularly those related to recruitment.

### Conclusions

Findings from our study will inform changes to the *we*Survive intervention’s conceptual model, program design, and recruitment and delivery protocols. The recommendations identified through our study will be incorporated into the next version of *we*Survive. Engagement in the participatory development process and initial proof-of-concept testing strengthens *we*Survive and will lead to the development of a behavioral intervention that could positively impact the health of cancer survivors in rural Appalachia and be more readily translated into practice. Importantly, the findings also stress the importance of using a model, such as the ORBIT framework, when developing or adapting behavioral interventions for cancer survivors. By conducting small-scale proof-of-concept studies, the feasibility of the novel or adapted intervention can be assessed relatively quickly and inexpensively, and the necessary revisions can be made before larger-scale testing.

## Abbreviations

CAB: Community Advisory Board
IRB: Institutional Review Board
L-CAT: Stanford Leisure-Time Activity Categorical Item
NCI: National Cancer Institute
ORBIT: Obesity-Related Behavioral Intervention Trials
PA: physical activity
QoL: Quality of Life
SCT: Social Cognitive Theory
SMART: specific, measurable, attainable, relevant, time-based
UVA: University of Virginia
